# Toxigenic diphtheria rarely detected amid rising cases of nontoxigenic *Corynebacterium diphtheriae* infections in Ontario, Canada, 2011–2023

**DOI:** 10.1128/jcm.01452-25

**Published:** 2025-12-04

**Authors:** Lisa R. McTaggart, Alexandra Scione, Kelty Hillier, Elizabeth Brown, Sarah E. Wilson, Julianne V. Kus

**Affiliations:** 1 Public Health Ontario 153300, Toronto, Ontario, Canada; 2 Dalla Lana School of Public Health, University of Toronto 7938, Toronto, Ontario, Canada; 3 Department of Laboratory Medicine and Pathobiology, University of Toronto 7938, Toronto, Ontario, Canada; Department of Medicine, Maine Medical Center, Portland, Maine, USA

**Keywords:** genomic epidemiology, toxin, public health, *Corynebacterium diphtheriae*, diphtheria

## Abstract

Diphtheria remains a major public health threat in areas of the world where diphtheria toxoid-containing vaccine programs are not successfully implemented or maintained. In countries with high vaccine coverage, diphtheria is well-controlled with occasional travel-related cases eliciting a robust public health response to limit transmission. While classic diphtheria is caused by toxigenic *Corynebacterium diphtheriae* and rarely by other species, such as *Corynebacterium ulcerans*, infections due to nontoxigenic *C. diphtheriae* (not covered by the vaccine) are increasingly being reported. We describe the genomic epidemiology of toxigenic and nontoxigenic *C. diphtheriae* in Ontario, Canada, based on isolates submitted to the Public Health Ontario Laboratory. Of 146 cases, four cases (2.7%) involved toxigenic *C. diphtheriae*, three of which were associated with travel from endemic countries. Most isolates were nontoxigenic (97.2%) and from cutaneous sources (84.9%), with an increase in submission of nontoxigenic *C. diphtheriae* isolates from one in 2016 to 65 in 2023. Based on whole-genome sequencing and BIGSdb-Pasteur core genome multi-locus strain typing, most nontoxigenic isolates were categorized into three highly related genetic clusters from sublineages 32 and 76, suggesting epidemiologically linked cases indicative of local transmission. Apart from intermediate susceptibility to penicillin in 98.0% of isolates, antimicrobial resistance was rarely detected. Because infections with nontoxigenic *C. diphtheriae* are not prevented by vaccination and do not cause classic diphtheria, healthcare professionals should be aware of these trends, given the clinical, public health, and infection control implications when *C. diphtheriae* is identified in the microbiology laboratory prior to knowledge of toxigenicity.

**IMPORTANCE:**

Infections by toxigenic and nontoxigenic *Corynebacterium diphtheriae* cause vastly different diseases requiring different treatment and public health responses. Healthcare practitioners should be aware of the complexities of diphtheria from a global perspective. Toxigenic *C. diphtheriae* remains prevalent in regions that do not have well-established vaccination programs, while vaccine hesitancy and compliance issues challenge countries that do require childhood vaccination. These factors, coupled with the propensity for human travel and migration, heighten the risk of cases and outbreaks in non-endemic countries and designate diphtheria as a relevant re-emerging threat. Further complicating public health decisions is the increasing incidence of nontoxigenic *C. diphtheriae* infections identified among highly vaccinated populations. An excessive public health response in these cases would be burdensome and expensive. Our findings will aid public health departments and hospitals with local risk assessments of the likelihood that a laboratory identification of *C. diphtheriae* represents a case of toxigenic diphtheria by raising awareness of the disproportionately small number of toxigenic *C. diphtheriae* recovered from clinical specimens compared to the vast majority of nontoxigenic isolates. Genomic analysis suggests local transmission of nontoxigenic strains, while isolation of toxigenic *C. diphtheriae* from among a highly vaccinated population remains associated with travel to endemic regions.

## INTRODUCTION

Diphtheria is a highly contagious infectious disease with classic manifestations as an acute respiratory infection often including the formation of a toxin-induced, suffocating pseudomembrane in the throat and nose, and a high case fatality rate (5%–17%) (1). When there is a strong clinical suspicion of diphtheria, prompt administration of diphtheria antitoxin is needed to neutralize the diphtheria toxin (DT). Primarily caused by strains of *Corynebacterium diphtheriae* carrying the prophage-encoded DT, the disease can also be caused by *Corynebacterium belfantii* (2) and the zoonotic pathogens *Corynebacterium ulcerans, Corynebacterium ramonii,* and *Corynebacterium pseudotuberculosis* if they acquire a functional DT gene (1, 3, 4). Because of the highly effective, widely used DT vaccine, diphtheria is considered a disease of the past. Cases of toxigenic diphtheria remain rare in countries with high vaccine coverage, although sporadic cases are detected among travelers from countries where diphtheria is endemic due to low vaccination coverage (1, 5). While classical respiratory diphtheria remains the primary public health concern, the epidemiology of *C. diphtheriae* infections is changing with an increasing number of cutaneous infections caused by nontoxigenic strains (6–10). Cases of cutaneous infection are identified when nontoxigenic *C. diphtheriae* is isolated from wounds also containing *Staphylococcus aureus*, *Streptococcus pyogenes,* or *Arcanobacterium haemolyticum* (8, 11–13). Unimpeded by the DT vaccine, nontoxigenic *C. diphtheriae* appear to circulate among people who are unstably housed and/or experiencing substance use disorders (6–8, 10). Given the increase in reported cases of nontoxigenic *C. diphtheriae* and the potential for cutaneous infections to develop into serious invasive disease, such as endocarditis and bacteremia, some consider it an emerging pathogen (8, 12).

Understanding the epidemiology of both toxigenic and nontoxigenic *C. diphtheriae* infections is important as it can help inform local risk assessments regarding treatment and appropriate public health and infection control responses, especially while toxigenic testing is pending following laboratory confirmation of *C. diphtheriae* from a clinical specimen. Combining genomics with epidemiological data enhances surveillance and global tracking of pathogen transmission. Genomic diversity of circulating strains suggests extensive global transmission of toxigenic and nontoxigenic *C. diphtheriae,* with asymptomatic or cutaneous carriage likely serving as an important component of transmission chains (5, 11, 14, 15). In cases of diphtheria, genomic surveillance and tracking of toxigenic *C. diphtheriae* strains can elucidate the acquisition source for infection control purposes and enable monitoring of the *tox* gene for mutations that could potentially decrease toxoid vaccine antigenic match and efficacy (9). Whole-genome sequencing (WGS) and an established core genome multi-locus sequence typing scheme (cgMLST) (16) allow for enhanced strain typing amenable to cross-regional comparison to facilitate strain tracking and an understanding of genomic epidemiology and genetic diversity of *C. diphtheriae*. WGS also enables the *in silico* detection of antimicrobial resistance (AMR) genes to better understand the dynamics and spread of genetic determinants of resistance.

In this study, we describe the epidemiology of toxigenic and nontoxigenic *C. diphtheriae* from 2011 to 2023 in Ontario, Canada’s most populous province, representing 15.6 million people (17). Beyond documenting temporal changes in incidence and regional variations in disease occurrence, we use cgMLST to contextualize study isolates within the global diversity of strains and to uncover cryptic epidemiological linkages suggestive of local transmission chains. Together with phenotypic antimicrobial susceptibility testing data and scans for AMR genes, we contribute genomic analysis from toxigenic and nontoxigenic isolates from a North American jurisdiction to enrich the understanding of the global epidemiology of *C. diphtheriae*.

## MATERIALS AND METHODS

As per provincial guidelines, all *C. diphtheriae* isolates are submitted to Public Health Ontario (PHO) Laboratory, Ontario’s reference microbiology laboratory, for identification confirmation by matrix-assisted laser desorption/ionization-time-of-flight mass spectrometry (MALDI-ToF MS) and ability to reduce nitrate, antimicrobial susceptibility testing (AST), and toxigenicity determination by DT PCR and modified-ELEK immunoprecipitation performed by the National Microbiology Laboratory-Public Health Agency of Canada.

For the study period (1 January 2011 to 31 December 2023), laboratory data were linked with data extracted from Ontario’s provincial surveillance system used for reportable diseases (Integrated Public Health Information System) and information obtained through public health consultations (toxigenic isolates only) to inform case and contact management that occurred between PHO and local Public Health Units (PHUs) tasked with local delivery of public health programs for disease prevention. Case data included patient sex, age range, geographic location (PHU and geographic region), exposure (acquisition/transmission), travel history, and hospitalization. Geographic regions (North West, North East, Eastern, Central East, Central West, Toronto, and South West) were assigned based on the patient’s residential postal code or the submitter’s postal code if patient’s postal code was unknown.

Annual incidence or average annual incidence was calculated using the annual population or the 5-year average population (2019–2023), respectively, as denominators. Population estimates for Ontario and each PHU were obtained from Statistics Canada (17). Maps were generated using Easy Maps v2.0, developed by PHO.

AST to determine minimal inhibitory concentration (MIC) was performed by the broth microdilution method according to Clinical and Laboratory Standards Institute (CLSI) M45 (18) using Mueller-Hinton broth with lysed horse blood (5%) and the Sensititre Microbiology System with plate GPALL1F (TREK Diagnostic Systems, USA). Interpretative criteria (susceptible, intermediate, resistant) were applied to MIC values according to CLSI M100 (19).

WGS libraries were prepared using the Nextera XT library preparation kit (Illumina, San Diego, CA, USA) from DNA extracted from cryopreserved isolates using the NucliSens eMAG System (bioMérieux, Marcy-l’Étoile, France). Libraries were sequenced on the Illumina NextSeq550 mid-output, 300-cycle cartridges as per the manufacturer’s protocol (Illumina). Data are available under BioProject PRJNA1209833.

*De novo* assembled genomes were uploaded to the BIGSdb-Pasteur platform (https://bigsdb.pasteur.fr/diphtheria/) for cgMLST (16). Concatenated cgMLST sequences were downloaded and used for phylogenetic analysis using IQTREE-2 (20), with branch lengths corrected to account for recombination using ClonalFrameML v1.12 (21). For select sublineages (SL), minimum-spanning trees contextualizing study isolates with other isolates of the same SL were visualized using the BIGSdb-Pasteur platform. Genetic markers for AMR were identified using the Resistance Gene Identifier (RSI) v6.0.3 with the Comprehensive Antimicrobial Resistance Database (CARD) v3.3.0 (22). Additionally, we used BLASTN (identity >80%, coverage >90%) to search genomes for the presence of the *tox* gene sequences of strain PW8 (23) and penicillin-binding protein *pbp2m* (24).

## RESULTS

From 2011 to 2023, there were 159 isolates submitted to the PHO laboratory that were identified as *C. diphtheriae* by MALDI-ToF MS. However, subsequent biochemical testing identified six isolates as *C. belfantii* based on their inability to reduce nitrate. Excluding *C. belfantii*, there were 146 cases, represented by 153 isolates (duplicate isolates were submitted for seven cases), of culture-confirmed infections caused by *C. diphtheriae* in Ontario.

### Toxigenic *C. diphtheriae*

**Fig 1:**
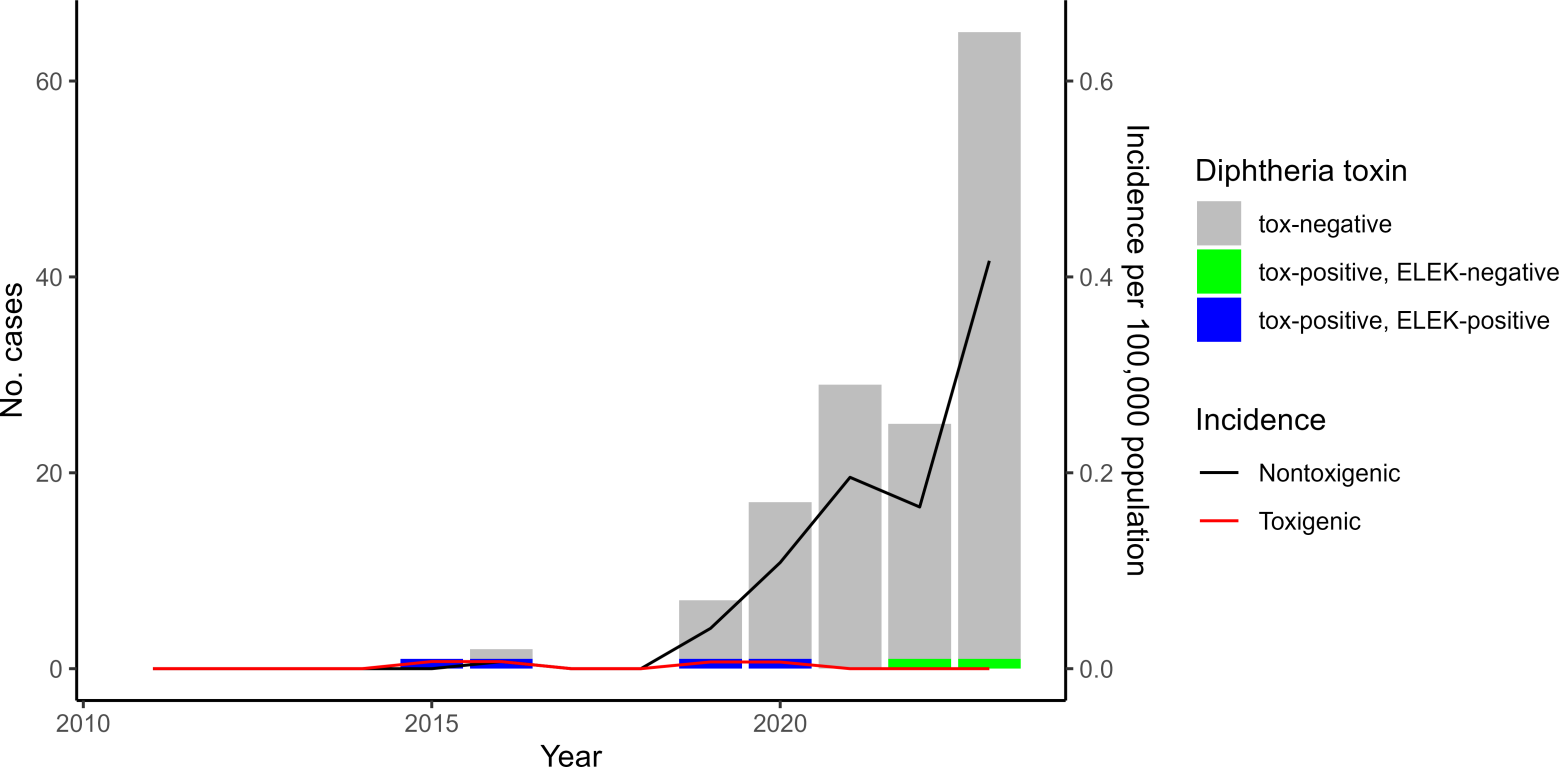
Number and incidence of toxigenic (*tox*-positive, ELEK-positive) and nontoxigenic (*tox*-positive, ELEK-negative, and *tox*-negative) *C. diphtheriae* in Ontario, 2011–2023.

Of 146 cases, only four cases (2.7%) were caused by toxigenic (*tox*-positive, ELEK-positive) isolates (Fig. 1). Cases involving *tox*-positive isolates were temporally disparate, with single cases detected in 2015, 2016, 2019, and 2020. Yearly incidence remained stably low, ranging from 0 to 0.007 per 100,000 population per year (Fig. 1). Two toxigenic isolates were cultured from respiratory specimens (lung and sputum), and two from cutaneous sources (unspecified wound and foot wound). Diphtheria *tox* gene sequences of two respiratory isolates were identical to that of the vaccine strain PW8 (23), while the other two cutaneous isolates had *tox* gene sequences identical to historical strain 31A, possessing two synonymous mutations compared to the PW8 sequence (23).

Three of four cases involving toxigenic *C. diphtheriae* reported international travel prior to infection and cgMLST profiles substantiated potential travel-related acquisition. SL designation, defined as a group of isolates with at least 500 of 1,305 identical cgMLST alleles (16) matched the genetic profile of other isolates from the country or region of travel, namely a SL405 toxigenic isolate from a patient with travel to India, a SL421 isolate from a patient with travel to Madagascar, and a SL302 isolate from a patient with travel to Southeast Asia, specifically Indonesia (Table 1). One toxigenic isolate was recovered from the lung of a hospitalized individual with unknown travel history; it belonged to SL466, together with other toxigenic isolates from India or from migrants to Europe with documented travel from South Asia (Table 1). This patient was not classified as meeting the clinical criteria of diphtheria due to a lack of symptoms compatible with clinical illness.

**TABLE 1:** SLs and genetic clusters (GCs) as classified by the BIGSdb-Pasteur cgMLST scheme (16) of 144 Ontario *C. diphtheriae* isolates, together with the geographic location(s) and diphtheria *tox* gene PCR of other isolates of the same SL, travel histories, and laboratory test results for *tox* PCR and modified ELEK tests for DT

SL	Geographic location(s) of BIGSdb-Pasteur database SL isolates; *tox* gene PCR	GC	No. of Ontario isolates	Travel history	DT testing
Toxigenic *C. diphtheriae*					
SL466	India (*n* = 16), France via IR, TR, BG, RS, HU, AT, CH (*n* = 1), Switzerland (*n* = 1), Germany via SY (*n* = 1), Germany (*n* = 1), Germany via PK, IR, TR, EL, CH, AT (*n* = 1), Austria (*n* = 3); *tox*-positive	GC30	1	NA^*a*^	*tox*-positive, ELEK-positive
SL405	India (*n* = 13); *tox*-positive	GC1040	1	India	*tox*-positive, ELEK-positive
SL302	Malaysia (*n* = 1), France (*n* = 1), France via TH (*n* = 1), Australia (*n* = 3); *tox*-positive	GC1042	1	Indonesia	*tox*-positive, ELEK-positive
SL421	Réunion (*n* = 2), Madagascar (*n* = 1); *tox*-positive	GC1038	1	Madagascar	*tox*-positive, ELEK-positive
Nontoxigenic *C. diphtheriae*					
SL10034		GC1045	1	NA	*tox*-negative
SL308	France (*n* = 4), Austria (*n* = 1), India (*n* = 2), Germany (*n* = 1); *tox*-positive/negative	GC1039	1	NA	*tox*-negative
SL301	India (*n* = 7), Austria (*n* = 2), Australia (*n* = 1), Syria (*n* = 1); *tox*-positive	GC1053	1	NA	*tox*-positive, ELEK-negative
SL422	France (*n* = 2), Spain (*n* = 1); *tox*-negative	GC376	1	NA	*tox*-negative
SL5	Australia (*n* = 2), Germany (*n* = 1), Russia (*n* = 1), France (*n* = 6), Austria (*n* = 1), Spain (*n* = 1), Romania (*n* = 1), Belarus (*n* = 37), Italy (*n* = 1); *tox*-positive/negative	GC23	2	NA	*tox*-negative
SL990		GC1046	1	Egypt	*tox*-negative
SL717	France (*n* = 1); tox-negative	GC1052	4	NA	*tox*-negative
SL50	Mayotte (*n* = 4), Réunion (*n* = 1), France (*n* = 1); USA (*n* = 10), Canada (*n* = 67); *tox-*positive/negative	GC1049	1	NA	*tox*-positive, ELEK-negative
SL863		GC502	1	NA	*tox*-negative
SL76	Canada (*n* = 45), Belarus (*n* = 5), UK (*n* = 1); *tox*-negative	GC1041	46	NA	*tox*-negative
		GC1044	2	NA	
		GC1047	1	NA	
		GC1050	1	NA	
SL228	Australia (*n* = 2), France (*n* = 2), New Caledonia (*n* = 33), French Polynesia (*n* = 5), Romania (*n* = 1); *tox*-negative	GC845	1	NA	*tox*-negative
SL295	Eritrea (*n* = 1), France via SL (*n* = 3), Yemen (*n* = 2), Austria (*n* = 1), Germany via LK (*n* = 4), India (*n* = 2); *tox*-negative	GC1048	1	NA	*tox*-negative
SL441	France (*n* = 1), Germany (*n* = 2), Canada (*n* = 1); tox-negative	GC1054	1	NA	*tox*-negative
SL542	France via SN (*n* = 1); Spain (*n* = 1), *tox*-negative	GC1051	1	Sri Lanka	*tox*-negative
		GC503	1	NA	
SL32	Australia (*n* = 5), Spain (*n* = 4), Austria (*n* = 2), France (*n* = 8), Russia (*n* = 2), UK (*n* = 1), Italy (*n* = 2), Belarus (*n* = 2), Romania (*n* = 1); *tox*-negative	GC1043	42	NA	*tox*-negative
		GC28	30	NA	

^*a*^ NA: not available.

### Nontoxigenic *C. diphtheriae*

The majority of *C. diphtheriae* isolates were nontoxigenic (*n* = 142, 97.2%), either *tox* gene-negative (*n* = 140) or nontoxigenic toxin-bearing (NTTB) isolates (*n* = 2, 1.4%), which were *tox* gene-positive but ELEK-negative due to internal stop codons in the *tox* gene sequences. Cases involving nontoxigenic isolates were identified in all age groups (median = 41 years old), with the majority from 20 to 39 (*n* = 51, 35.9%) and 40 to 59 (*n* = 52, 36.6%) year-olds; 57.7% were from males. Most cases were from cutaneous sources (*n* = 122, 85.9%), but four cases (2.8%) were from respiratory specimens and four (2.8%) were from blood.

**Fig 2:**
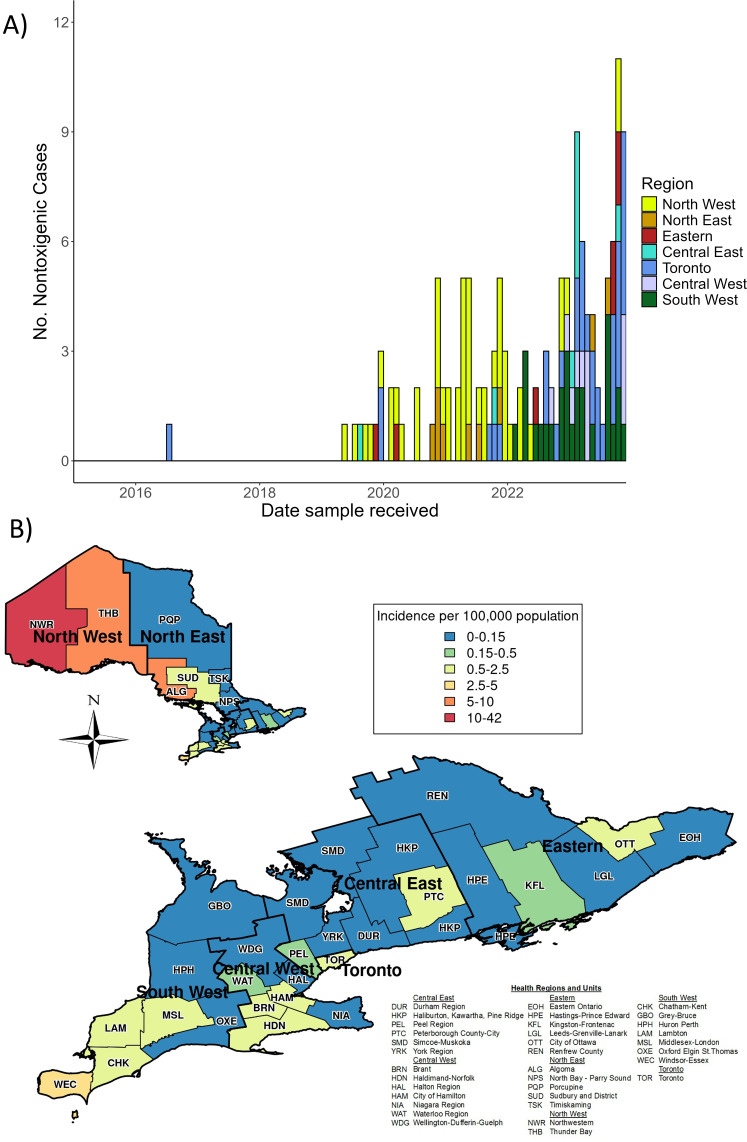
(**A**) Number of cases of nontoxigenic *C. diphtheriae* in seven regions of Ontario, aggregated by month, 2016–2023. (**B**) Average annual incidence (2019–2023) of cases of nontoxigenic *C. diphtheriae* infections in Ontario’s 34 Public Health Units, categorized regionally as in panel **A**.

Known cases of nontoxigenic *C. diphtheriae* infections in Ontario increased 6,400% from one case in 2016 (0.007 per 100,000 population) to 65 cases in 2023 (0.416 per 100,000 population) (Fig. 1). The greatest proportion was detected in the North West region of the province (*n* = 47, 33.1%), followed by Toronto (*n* = 33, 23.2%) and the South West (*n* = 27, 19.0%). Based on the temporal distribution of cases, the North West region had the highest proportion of cases between 2019 and 2021 (2019 = 4/6, 66.6%; 2020 = 10/16, 62.5%; 2021 = 21/29, 72.4%), the South West region had the highest proportion in 2022 (10/25, 40.0%), and Toronto had the highest proportion in 2023 (23/65, 35.4%) (Fig. 2A). Due to differences in population sizes, the North West region had the highest average annual incidence rate during the final 5 years of the study from 2019 to 2023 (Fig. 2B).

**Fig 3:**
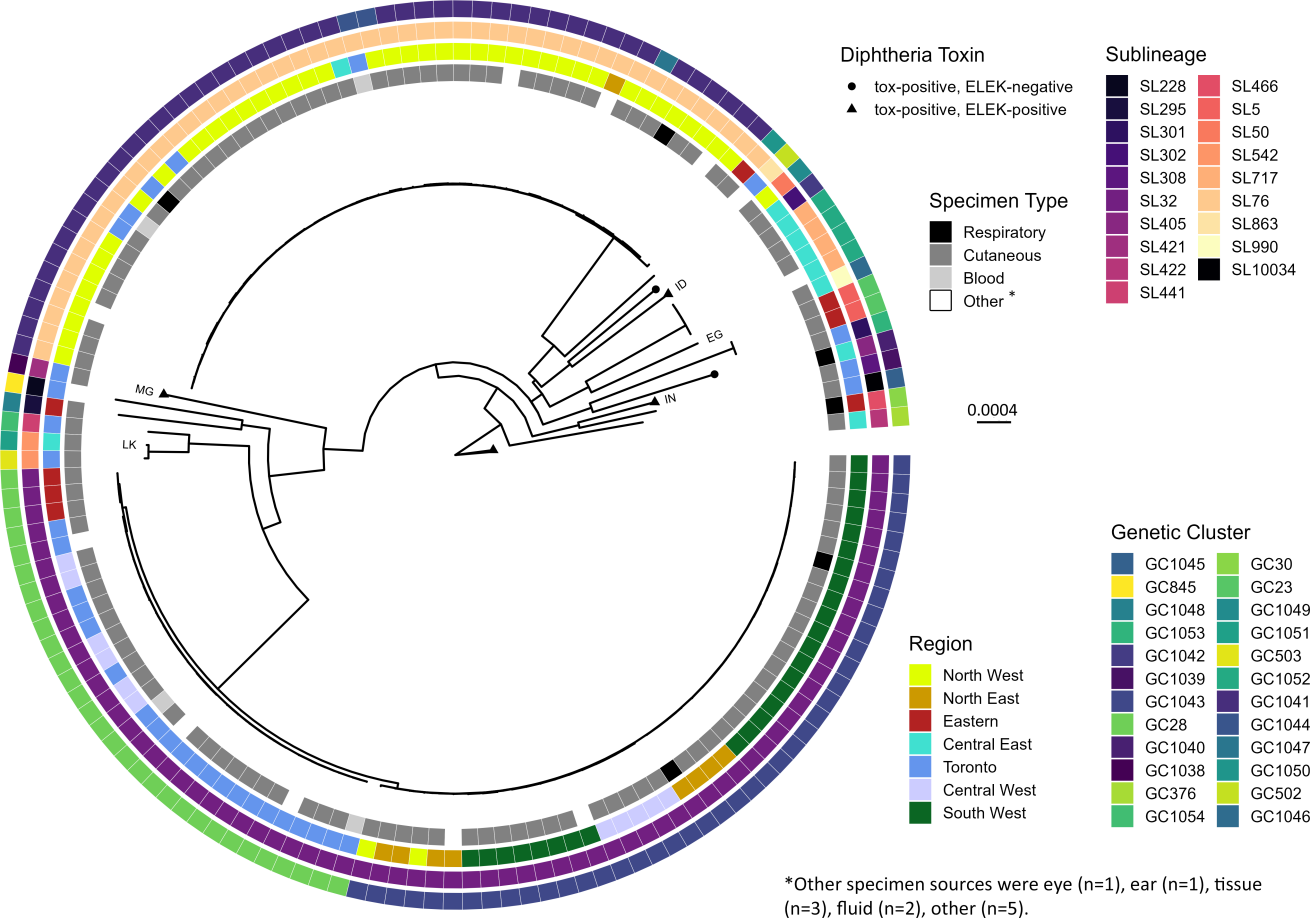
Phylogeny of concatenated cgMLST gene sequences from 144 Ontario *C. diphtheriae* isolates. Branch tips denote the detection of *tox* gene and ELEK result. Cases with known travel history (*n* = 5) to India (IN), Egypt (EG), Sri Lanka (LK), Madagascar (MG), and Indonesia (ID) are noted. Metadata circles from inside to outside refer to specimen type, geographic region, SL, and GC.

**Fig 4:**
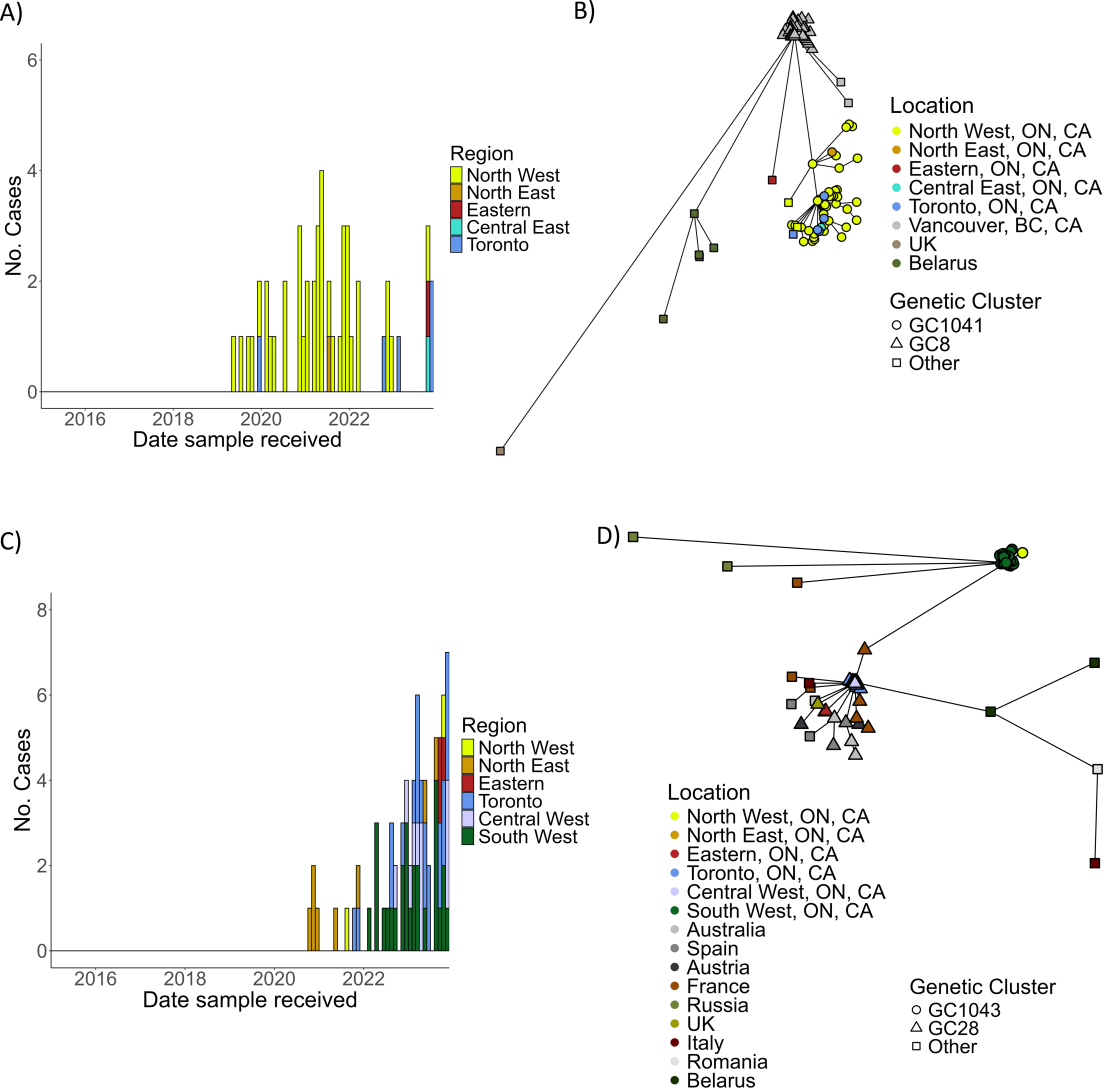
Number of cases of SL76 (**A**) and SL32 (**C**) by region in Ontario, aggregated by month, 2015–2023. Minimum spanning trees based on cgMLST of SL76 (**B**) and SL32 (**D**), including isolates from Ontario and reference isolates retrieved from BIGSdb-Pasteur. SL76 isolates were primarily GC1041 (circle) from Ontario or GC8 (triangle) from Vancouver. SL32 isolates were primarily either GC1043 (circle) or GC28 (triangle).

Including four cases of toxigenic diphtheria (above) and 140 cases of nontoxigenic *C. diphtheriae* infection (two isolates were unavailable for WGS) cgMSLT and phylogenetic analysis confirmed all isolates as *C. diphtheriae* sensu stricto and classified them into 19 SLs (Table 1; Fig. 3). Several isolates, including four toxigenic isolates and five travel-associated isolates (three toxigenic and two nontoxigenic), were genetically unique; a SL contained a single isolate, although these SLs were also represented by isolates from other countries (Table 1; Fig. 3). A large proportion of Ontario nontoxigenic isolates were classified into SL76 (*n* = 50; 34.7%) or SL32 (*n* = 72; 50.0%) (Fig. 3). SL76 isolates were primarily derived from cases from the North West region of the province recovered between June 2019 and December 2023 (Fig. 4A). According to the BIGsdb-Pasteur database, SL76 isolates were also recovered from cases in Vancouver, Canada (*n* = 45) (BIGsdb-Pasteur IDs 855, 880, 904, 905, 1084–1092, 1094–1125), UK (*n* = 1) (BIGsdb-Pasteur ID 553), and Belarus (*n* = 5) (BIGsdb-Pasteur IDs 198, 200, 232, 236, 271). Isolates were further classified into genetic clusters (GC) that differed by less than 25 of 1,305 cgMLST alleles. Most Ontario SL76 isolates (*n* = 46; 92.0%) were highly similar, forming GC1041 and genetically distinct from GC8, which comprised 95.6% (*n* = 43) of the Vancouver isolates (Fig. 4B). SL32 isolates were recovered from multiple regions of Ontario between November 2020 and December 2023 and also from Australia (*n* = 5) (BIGsdb-Pasteur ID 82, 117, 912, 922, 923), Spain (*n* = 4) (BIGsdb-Pasteur ID 1314, 1315, 1322, 1330), Austria (*n* = 2) (BIGsdb-Pasteur ID 1226, 1233), France (*n* = 11) (BIGsdb-Pasteur ID 357, 373, 812, 897, 900, 1083, 1483, 1490, 2232, 3377, 3629), Russia (*n* = 2) (BIGsdb-Pasteur ID 316, 603), UK (*n* = 1) (BIGsdb-Pasteur ID 29), Italy (*n* = 2) (BIGsdb-Pasteur ID 34, 35), Belarus (*n* = 2) (BIGsdb-Pasteur ID 248, 268), and Romania (*n* = 1) (BIGsdb-Pasteur ID 816) (Fig. 4C and D). Ontario SL32 isolates were divided into either GC1043, comprising isolates from the South West, North East, and North West regions from November 2020 to December 2023, or GC28 consisting of isolates from Toronto, Central West, and Eastern regions from November 2021 to December 2023 together with isolates from Austria, Australia, Spain, France, and the UK (Fig. 4D).

AST was performed on 49 nontoxigenic *C. diphtheriae* isolates representing unique cases. A single isolate was resistant to clindamycin and tetracycline, while two isolates were resistant only to tetracycline (Table S1, Fig. S1). The majority (98.0%) of tested isolates displayed intermediate susceptibility to penicillin based on current CLSI interpretations (25) (Table S1). Genomes (*n* = 144) were interrogated using the RSI with CARD (22) to detect genes known to confer AMR. The isolate resistant to both clindamycin and tetracycline possessed *ermX* and *tet(33); tet(O*) was present in the genomes of two tetracycline-resistant isolates (Fig. S1). Several other genes known to confer AMR in other bacteria were detected, including *APH(3′)-Ia*, *APH(3″)-Ib*, and *APH(6)-Id*, *tet(W*), a D95G *gyrA* mutation, *sul1*, and *cmx*. Penicillin resistance gene *pbp2m(24)* was not detected in any isolate (Fig. S1).

## DISCUSSION

Here, we provide a timely update on the incidence, regional distribution, toxigenicity, antimicrobial susceptibility, and strain typing of *C. diphtheriae* infections in Ontario, Canada. Since provincial guidelines stipulate that all *C. diphtheriae* isolates must be submitted to PHOL for DT testing, this study collection likely represents high ascertainment of cases where *C. diphtheriae* was identified for this North American jurisdiction. Cases of nontoxigenic *C. diphtheriae* infections in Ontario increased by 6,400% in recent years. While the isolation of *C. diphtheriae* from a clinical specimen can be alarming, we emphasize that cases of toxigenic diphtheria in Ontario remained extremely low at 0 or 1 case per year and are highly associated with importation from endemic areas. Rather, the escalation in cases of *C. diphtheriae* infections was due to a surge in nontoxigenic *C. diphtheriae* from 2019 to 2023. It is possible that this increase was due in part to changes in laboratory detection methods and algorithms. With access to easy, inexpensive, and accurate bacterial identification systems like MALDI-ToF MS, some laboratories now identify all bacterial isolates from cutaneous sources, whereas previously many of these isolates would have been reported out as “diphtheroids” or “normal skin flora,” and *C. diphtheriae* were unknowingly missed (1). However, given the outbreaks of toxigenic diphtheria recently reported in many countries (1, 14, 26–30), as well as the increasing number of reports globally of nontoxigenic *C. diphtheriae* infections (6, 8–10, 12, 27, 31), further investigation of the genomic epidemiology of *C. diphtheriae* in Ontario is warranted to understand transmission of toxigenic and nontoxigenic strains.

Although only four cases of toxigenic *C. diphtheriae* were detected in Ontario from 2011 to 2023, understanding the mode of acquisition is vital to controlling the spread of disease. In countries with high vaccination rates, acquisition of diphtheria in travelers from countries where it is endemic is well-documented and substantiated by genetic profiling (5, 12, 14, 29). According to the BIGsdb-Pasteur platform, the cgMLST profiles of the isolates (SL405, SL421, SL302) were consistent with the epidemiological history of travel of the patients to India, Madagascar, and Southeast Asia (Indonesia). These findings suggest that in Ontario, Canada, travel history is an important criterion in a public health risk assessment for assessing the likelihood that a laboratory identification of *C. diphtheriae* represents a case of toxigenic diphtheria prior to the availability of the toxigenicity test results. However, a fourth case of toxigenic *C. diphtheriae* occurred in a patient with an unknown travel history and did not meet the clinical criteria for diphtheria. The cgMLST profile (SL466) from this case was genetically related to isolates (SL466 = ST466) from South Asia, postulated to have enhanced dissemination capabilities (26). Asymptomatic or sub-clinical carriage of *C. diphtheria*e in vaccinated populations is an important component of transmission chains (11, 14) and may have played a role in this case.

High efficacy (96%–98%) of the DT vaccine successfully maintains a low incidence of toxigenic diphtheria in populations with high vaccination coverage (1). However, vaccine escape could occur if mutations accumulate in the *tox* that decrease antigenic match. With 18 documented allelic variants, including eight non-synonymous single-nucleotide polymorphisms, *tox* diversity projects the genuine likelihood of vaccine escape. Accordingly, routine *tox* sequence surveillance is prudent (9). Toxigenic *C. diphtheriae* in Ontario exhibited only synonymous *tox* gene mutations. In addition, two Ontario isolates (SL50 and SL301) possessed the *tox* gene but failed to express it due to internal stop codons. *C. diphtheriae* NTTB isolates have previously been detected in Canada (7% of *C. diphtheriae* isolates) (32) and elsewhere (5%–20% of *tox*-positive isolates) (33). One hypothesis is that they have evolved due to a lack of selection pressure to maintain a functional *tox* gene during transmission in a highly vaccinated population (34).

The majority of *C. diphtheriae* (97.2%) in this study were nontoxigenic or NTTB isolates. Often isolated from wounds co-infected with *S. aureus, S. pyogenes,* or *A. haemolyticum* (8, 11–13), the contribution of nontoxigenic *C. diphtheriae* to disease pathology in this circumstance is unknown. However, nontoxigenic *C. diphtheriae* infections can be invasive, and several case reports describe endocarditis arising from nontoxigenic *C. diphtheriae* bacteremia (8, 12). Four nontoxigenic cases in our study (2.7%) were identified from blood, although no additional clinical information was available to determine if there were other manifestations of invasive disease.

Similar to studies from other countries (5, 9, 12, 14), *C. diphtheriae* isolates in Ontario were surprisingly diverse, the genetic profile and stochastic frequency of which suggest travel-related acquisition but cannot rule out the possibility of broad geographic (intercontinental) distribution of certain SLs (9). The majority of isolates clustered into one of three GCs representing two SLs (GC1041 of SL76, GC1043 of SL32, and GC28 of SL32), suggesting local transmission. Ontario SL76 isolates (GC1041) were genetically distinct from the SL76 isolates previously identified from cutaneous specimens from patients in a low-income urban population in Vancouver, B.C., Canada, 2015–2018 (GC8) (6). We hypothesize transcontinental importation of SL76 from Vancouver to Northwestern Ontario prior to detection in 2019, followed by local spread in the region. West-to-east interprovincial spread of pathogens, such as *emm*74 group A *Streptococcus,* has previously been documented (35). Diminished or delayed access to health care in rural and/or remote communities of Northwestern Ontario compared to the rest of the province (36) may have exacerbated local transmission in the region. SL32 isolates, the predominant SL of *C. diphtheriae* detected in Ontario, were classified into two GCs, suggesting extensive local transmission possibly following international acquisition since SL32 isolates were previously identified in Austria, Spain, Australia, France, and Russia. SL32 has been characterized as a virulent strain with superior adherence capacity due to the presence of all three pilus gene clusters in the genome (31), which likely enhanced the spread of this successful clone in Ontario.

Both toxigenic and nontoxigenic *C. diphtheriae* from cutaneous and respiratory sources have previously been detected in Canada (32, 37) and Ontario (38). Historically, reported detections were infrequent (13, 32), often with multi-year gaps (38), also manifest in our data (2011–2014, 2017–2018). Previous low numbers of reported nontoxigenic cases may be at least partially attributed to previous (pre-MALDI-ToF MS) laboratory practices in which “diphtheroids” from cutaneous specimens were rarely identified to species level, especially when other known pathogens (e.g., *S. pyogenes*, *S*. *aureus*) were co-isolated; some of the “diphtheroids” may have, in fact, been nontoxigenic *C. diphtheriae*. More recently, with the wide adoption of MALDI-ToF MS for bacterial identification, starting around 2013 in Canada, cases of nontoxigenic *C. diphtheriae* concurrently increased (37). This increase is possibly due to changing laboratory practices in which more cultured organisms are now identified from non-sterile sites where previously they would have been reported as “diphtheroids” or “normal skin flora” (37).

Concomitantly, local circulation of nontoxigenic strains has also been documented in a localized urban environment in Western Canada in recent years (1998–2007 [13], 2015–2018 [6]) and in Ontario, evidenced by the fact that 84.3% of isolates in this study belong to just three genetic clusters. So, while the surge in cases of nontoxigenic *C. diphtheriae* in Ontario (2019–2023) may be due to greater utilization of MALDI-ToF MS for more definitive bacterial identification in diagnostic laboratories, we cannot rule out the possibility that increased transmission of nontoxigenic strains also contributed to the recent proliferation of cases.

AMR of *C. diphtheriae* is minimal in many countries; however, AMR and even multidrug resistance have been detected in some regions (5, 9, 10, 12, 14, 24, 26, 31, 39). In Ontario, AMR among nontoxigenic *C. diphtheriae* remains low, and resistance to first-line antibiotics, specifically penicillin and erythromycin, was not detected. Two isolates with the tetracycline-resistant ribosomal protection protein gene *tet(O*) were resistant to tetracycline. One clindamycin and tetracycline-resistant isolate possessed *erm(X),* a gene encoding an rRNA methyltransferase that protects the ribosome from inactivation due to antibiotic binding, and *tet(33)*, the gene for a tetracycline efflux pump. Additional genes associated with AMR to several classes of antibiotics in other bacteria were identified, including aminoglycoside phosphotransferase genes [*APH(3′)-Ia*, *APH(3″)-Ib*, and *APH(6)-Id*], tetracycline-resistant ribosomal protection protein genes [*tet(W)*], a *gyrA* mutation causing a D95G substitution with potential to confer resistance to fluoroquinolone, sulfonamide-resistant dihydropteroate synthase gene (*sul1*), and chloramphenicol exporter gene *cmx,* although there were no clear phenotypic correlations in isolates investigated in this study. AMR genes, often associated with mobile elements, have previously been detected in *C. diphtheriae* (5, 9, 12, 24, 31, 40). Based on AMR surveillance, the number and breadth of AMR genes is greater in the last several years than in previous decades, prompting renewed vigilance (9).

Although none of the Ontario nontoxigenic *C. diphtheriae* isolates were resistant to penicillin, 98% displayed intermediate susceptibility according to CLSI breakpoints (S ≤ 0.12, R > 4) and 100% by EUCAST (S ≤ 0.001/R > 1) breakpoints (25, 41). This finding is not unique to Ontario isolates. Studies comparing penicillin MIC distribution patterns with CLSI or EUCAST breakpoints suggest the interpretation for virtually all wild-type *C. diphtheriae* isolates is intermediate susceptibility, possibly requiring increased or prolonged drug exposure due to inherent reduced activity against wild-type isolates (24, 39, 41). Of note, the *pbp2m* gene, known to confer low-level resistance to penicillin (24), was not detected among any Ontario *C. diphtheriae*.

There are limitations to our study. Incidence of infection may be underestimated since asymptomatic cases and self-resolving cases of nontoxigenic *C. diphtheriae* were not included. Vaccination status of patients, particularly in cases of toxigenic diphtheriae, was not known. Information on the clinical severity of nontoxigenic *C. diphtheriae* infections needed to fully appreciate disease morbidity was not known. Finally, sociodemographic risk factors, such as homelessness, drug or alcohol use, and unsanitary, overcrowded living conditions known to facilitate transmission of nontoxigenic *C. diphtheriae* (5–8, 10) were not assessed.

In conclusion, cases of toxigenic diphtheria were extremely low in Ontario. Only four cases were identified over 13 years, usually associated with travel from endemic countries. In contrast, nontoxigenic *C. diphtheriae* infections, often from cutaneous sources, increased 6,400% in recent years. While enhanced laboratory detection algorithms likely influenced the surge in detections of cases of nontoxigenic *C. diphtheriae*, genetic analysis also alluded to substantial local transmission of three GCs representing two different SLs. Almost all nontoxigenic isolates that were tested displayed intermediate susceptibility to penicillin, but they were rarely resistant to other antibiotics, although genomic analysis detected several AMR genes. Despite reassurances that the vast majority of *C. diphtheriae* isolated from clinical samples in Ontario are nontoxigenic, several factors advise appropriate vigilance regarding *C. diphtheriae* infections, including multiple recent diphtheria outbreaks reported globally, human travel-related transmission potentially facilitated by asymptomatic carriage, potential for decreased vaccine efficacy due to *tox* gene evolution, vaccine hesitancy, and the potential for nontoxigenic *C. diphtheriae* to cause serious invasive disease.

## Data Availability

Raw fastq data are available in BioProject PRJNA1209833.

## SUPPLEMENTAL MATERIAL

Table S1 and Figure S1 jcm.01452-25-s0001.pdf Table S1: Antimicrobial susceptibility data including range, MIC50, MIC90, percent susceptible (%S), percent intermediate susceptibility (%I), and percent resistant (%R) for nontoxigenic *C. diphtheriae* (n=49) isolates in Ontario. Figure S1: Phylogenetic distribution of antimicrobial susceptibility phenotypes and genes associated with antimicrobial resistance in bacteria. The phylogenetic tree from figure 3 is plotted with the CLSI interpretation of MICs from microbroth dilution panels (n=49 isolates) and the presence/absence of gene targets identified *in silico* by isolate genome interrogation with CARD-RSI or BLASTN searches (n = 144 isolates).

ASM does not own the copyrights to Supplemental Material that may be linked to, or accessed through, an article. The authors have granted ASM a non-exclusive, world-wide license to publish the Supplemental Material files. Please contact the corresponding author directly for reuse.
