# Supplementary material for: Toxigenic diphtheria rarely detected amid rising cases of nontoxigenic Corynebacterium diphtheriae infections in Ontario, Canada, 2011–2023
Source: J Clin Microbiol. 2025 Dec 4;63(12):e01452-25. doi: 10.1128/jcm.01452-25 (PMC12710362; doi:10.1128/jcm.01452-25)
Supplement: Table S1 and Figure S1 — Table S1: Antimicrobial susceptibility data including range, MIC50, MIC90, percent susceptible (%S), percent intermediate susceptibility (%I), and percent resistant (%R) for nontoxigenic C. diphtheriae (n=49) isolates in Ontario. Figure S1: Phylogenetic distribution of antimicrobial susceptibility phenotypes and genes associated with antimicrobial resistance in bacteria. The phylogenetic tree from figure 3 is plotted with the CLSI interpretation of MICs from microbroth dilution panels (n=49 isolates) and the presence/absence of gene targets identified in silico by isolate genome interrogation with CARD-RSI or BLASTN searches (n = 144 isolates). [file jcm.01452-25-s0001.pdf]

SUPPLEMENTAL MATERIAL

Table S1: Antimicrobial susceptibility data including range, MIC_{50}, MIC_{90}, percent susceptible (%S), percent intermediate susceptibility (%I) and percent resistant (%R) for nontoxigenic *C. diphtheriae* (n=49) isolates in Ontario.

	**Range**	**MIC_{50}**	**MIC_{90}**	**%S**	**%I**	**%R**
Ampicillin	<=0.12 - 1	0.5	0.5	NA	NA	NA
Chloramphenicol	<=2 - 16	<=2	<=2	NA	NA	NA
Ciprofloxacin	<=1	<=1 (S)	<=1 (S)	100.0%	0.0%	0.0%
Clindamycin	<=0.5 - >4	<=0.5 (S)	<=0.5 (S)	98.0%	0.0%	2.0%
Daptomycin	<=0.5 - 1	<=0.5 (S)	<=0.5 (S)	100.0%	NA	NA
Erythromycin	<=0.25 - 0.5	<=0.25 (S)	<=0.25 (S)	100.0%	0.0%	0.0%
Gentamicin	<=2	<=2 (S)	<=2 (S)	100.0%	0.0%	0.0%
Levofloxacin	<=0.25 - 0.5	<=0.25	<=0.25	NA	NA	NA
Linezolid	<=1	<=1 (S)	<=1 (S)	100.0%	NA	NA
Moxifloxacin	<=0.25 - 0.5	<=0.25	<=0.25	NA	NA	NA
Nitrofurantoin	<=32 - >64	64	>64	NA	NA	NA
Oxacillin	2 - >4	4	>4	NA	NA	NA
Penicillin	<=0.06 - 1	0.5 (I)	0.5 (I)	2.0%	98.0%	0.0%
Quinupristin-Dalfopristin	<=0.5	<=0.5	<=0.5	100.0%	0.0%	0.0%
Rifampin	<=0.5	<=0.5	<=0.5	100.0%	0.0%	0.0%
Streptomycin	<=1000	<=1000	<=1000	NA	NA	NA
Tetracycline	<=2 - >=32	<=2 (S)	<=2 (S)	93.9%	0.0%	6.1%
Tigecycline	<=0.03 - 0.5	<=0.03	0.06	NA	NA	NA
Trimethoprim-Sulfamethoxazole	<=0.5 - 1	<=0.5 (S)	<=0.5 (S)	100.0%	NA	0.0%
Vancomycin	<=0.25 - 1	0.5 (S)	0.5 (S)	100.0%	NA	NA

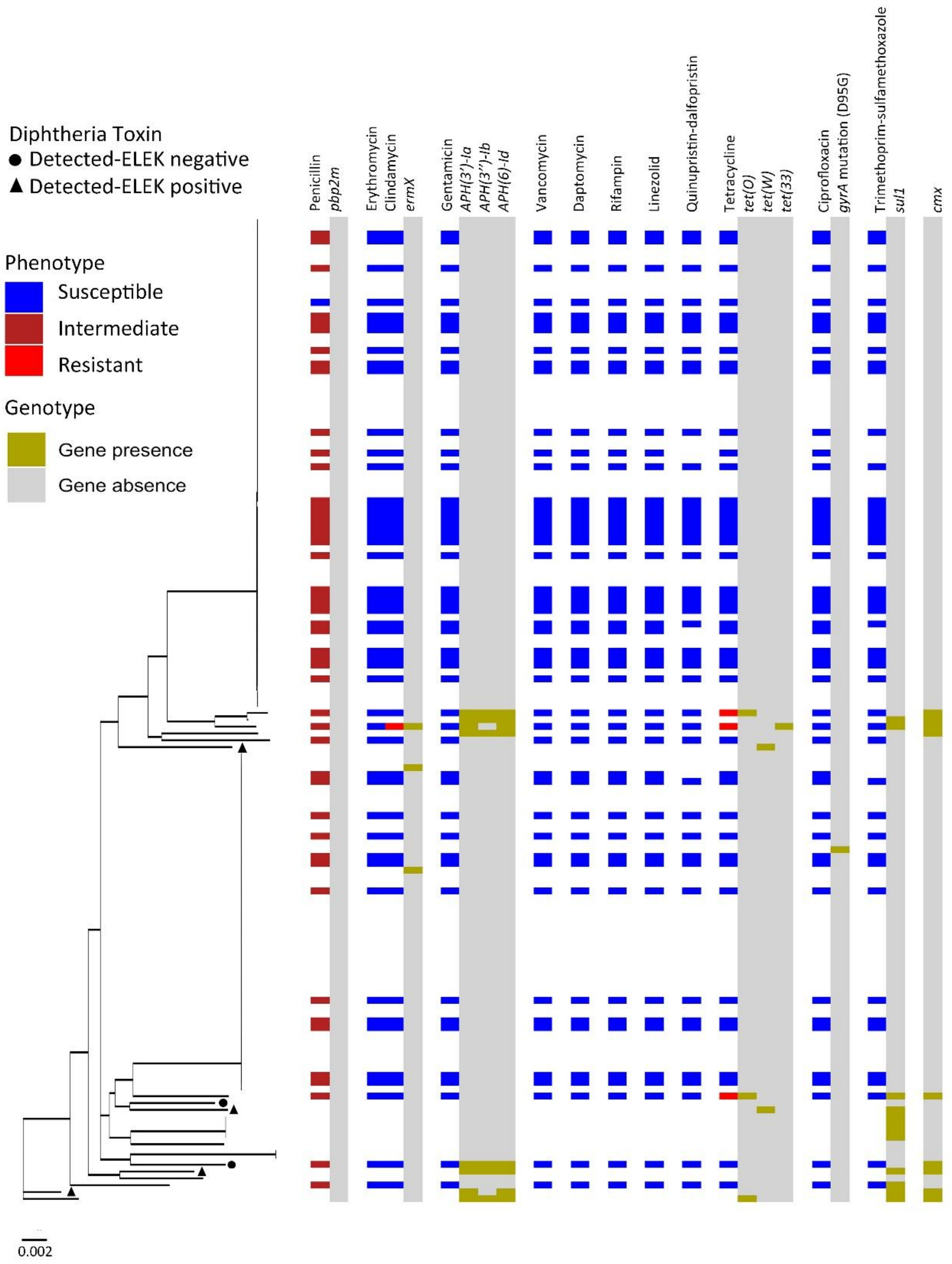

Figure S1: Phylogenetic distribution of antimicrobial susceptibility phenotypes and genes associated with antimicrobial resistance in bacteria. The phylogenetic tree from Figure 3 is plotted with the CLSI interpretation of MICs from microbroth dilution panels (n=49 isolates) and the presence/absence of gene targets identified in silico by isolate genome interrogation with CARD-RSI or BLASTN searches (n=144 isolates).
